# Rupture of Sinus of Valsalva Aneurysm into Interventricular Septum: Role of Cardiac CT

**DOI:** 10.7759/cureus.5589

**Published:** 2019-09-06

**Authors:** Abhishek Jain, Gayathri Achuthan

**Affiliations:** 1 Radiology, Columbia Asia Hospital, Pune, IND; 2 Radiology, Sahyadri Hospital, Pune, IND

**Keywords:** interventricular septum, ruptured sinus of valsalva aneurysm, aortic sinus, coronary arteries, cardiac ct

## Abstract

Sinus of Valsalva aneurysm dissecting and forming sinus tract into interventricular septum is an extremely rare complication of sinus of Valsalva aneurysm. Echocardiography and conventional angiography were used earlier to diagnose ruptured sinus of Valsalva aneurysm. Cardiac CT has emerged as a valuable non-invasive diagnostic tool for evaluation of complications of sinus of Valsalva aneurysm. In this article, we report two cases of ruptured sinus of Valsalva aneurysm arising from right and left coronary sinuses into the interventricular septum without aorto-cardiac shunt formation evaluated using 256 slice cardiac CT imaging. After diagnosis on cardiac CT, these findings were confirmed perioperatively and were repaired surgically.

## Introduction

Potentially fatal complications are seen with both ruptured and non-ruptured Valsalva sinus aneurysms; however, after treatment the prognosis is excellent [1]. Thus, it is important to make a timely diagnosis. Although most Valsalva sinus aneurysms are seen on echocardiography, electrocardiographically (ECG)-gated computed tomography (CT) can provide fine delineation of the relevant anatomy as well as associated complications.

This article depicts the role of cardiac CT in prompt and accurate diagnosis of interventricular septal rupture of sinus of Valsalva aneurysm, detailed depiction of sinus tracts and other complications as well as associated anomalies; thus, providing important piece of information to plan the further management.

## Case presentation

Case 1

A 20-year-old young male patient with past history of rheumatic heart disease presented with new onset chest pain, dyspnea and palpitations for 15 days.

Echocardiography revealed concentric left ventricular hypertrophy with severe (grade 4) mitral regurgitation and moderate aortic regurgitation. An aneurysmal cavity was detected at the base of inferior vena cava.

He was further evaluated with cardiac CT on a 256 slice CT scanner.

**Figure 1:**
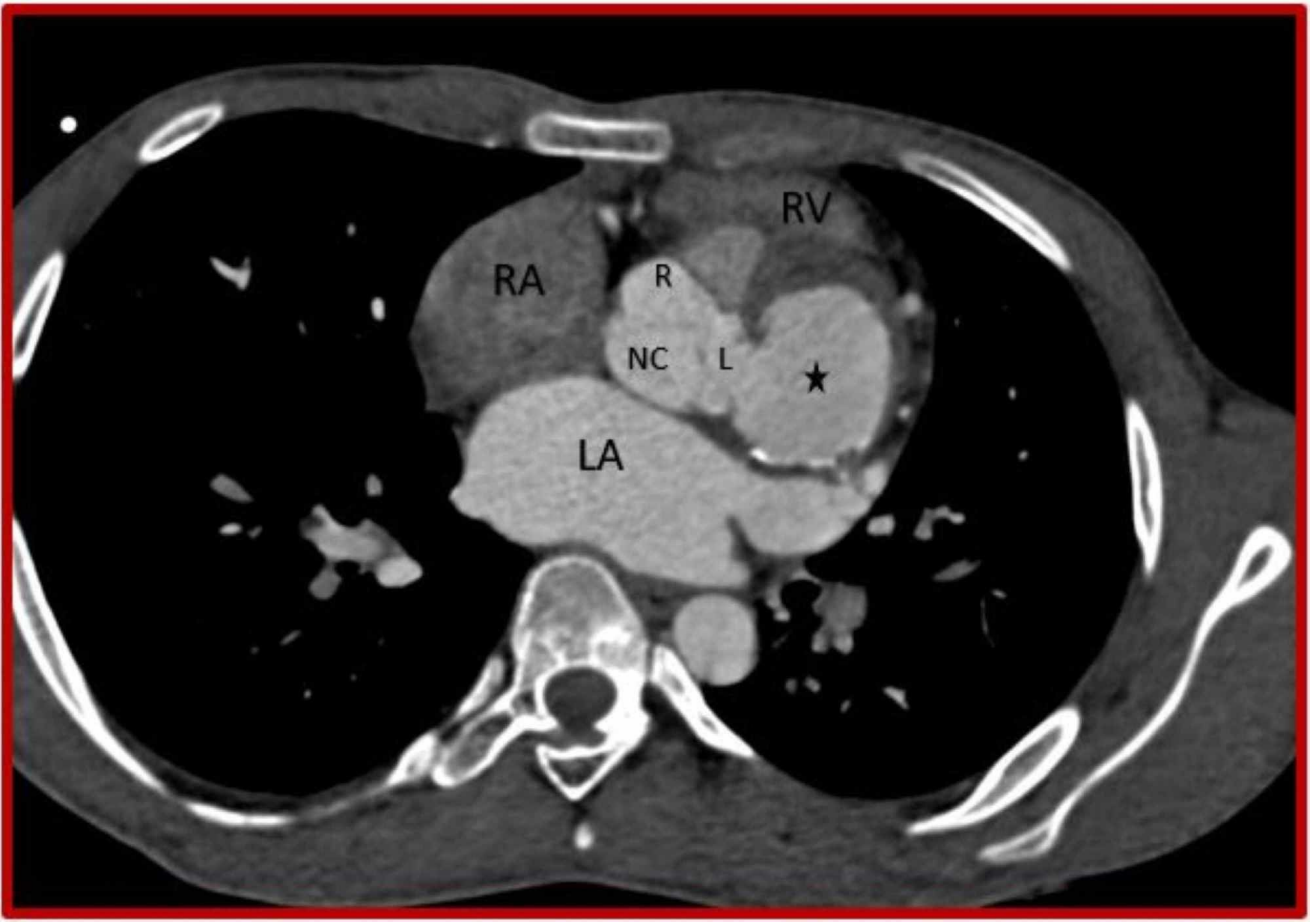
Cardiac CT (Axial view) demonstrates left sinus of Valsalva aneurysm (asterisk) with peripheral mural calcifications. CT: Computed tomography; RV: Right ventricle; RA: Right atrium; LA: Left atrium; R: Right coronary sinus; L: Left coronary sinus; NC: Non-coronary sinus.

A lobulated saccular aneurysm was seen arising from left coronary sinus with a narrow neck (Figure 1). The walls of the aneurysm showed curvilinear and coarse calcifications. No aneurysmal thrombosis was seen.

**Figure 2:**
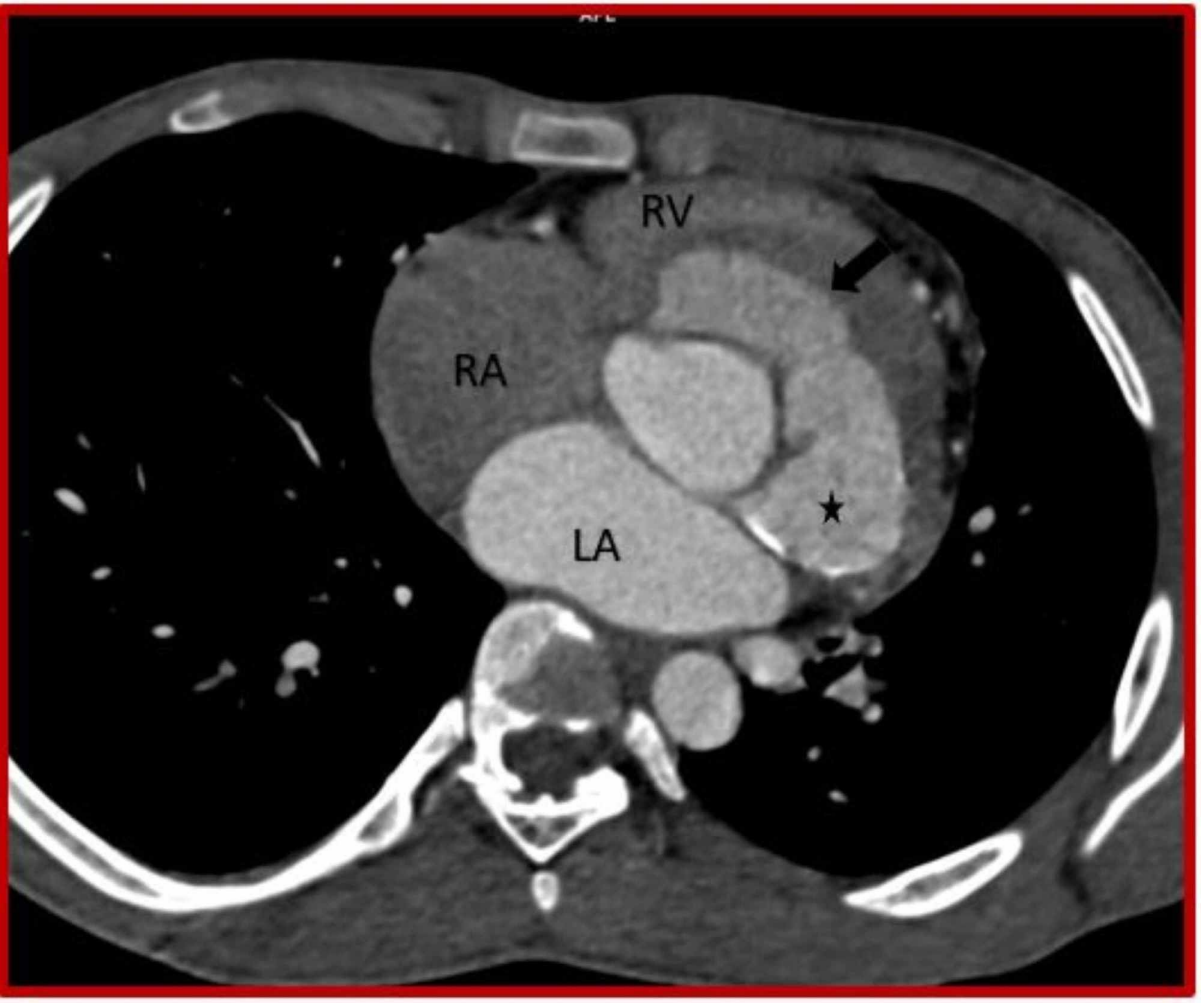
Cardiac CT (Axial view) demonstrates ruptured left sinus of Valsalva aneurysm (asterisk) dissecting into the interventricular septum (black arrow). CT: Computed tomography; RV: Right ventricle; RA: Right atrium; LA: Left atrium.

**Figure 3:**
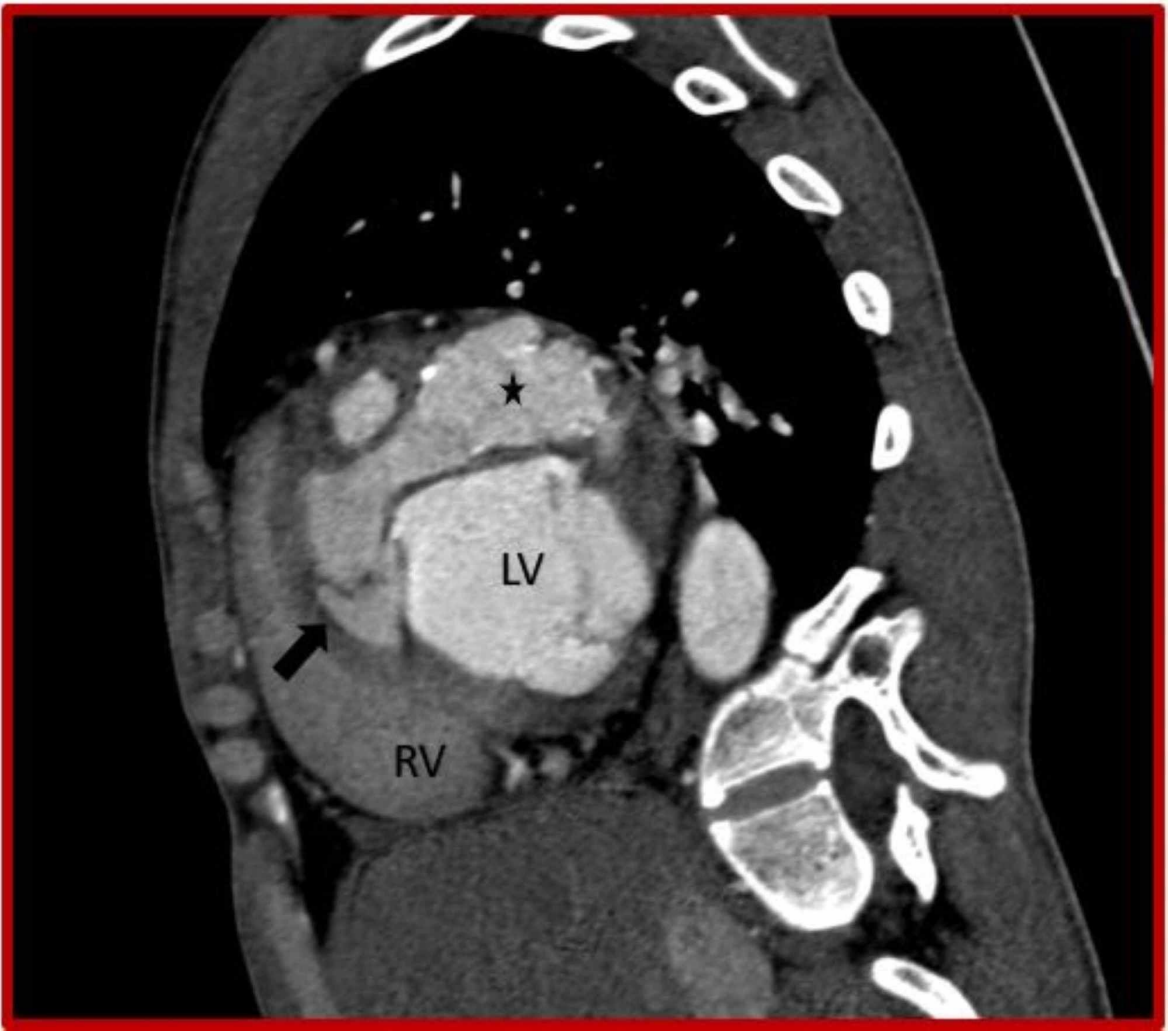
Cardiac CT (sagittal oblique view) demonstrates ruptured left sinus of Valsalva aneurysm (asterisk) dissecting into the interventricular septum (black arrow) from the basal to mid septal region. CT: Computed tomography; RV: Right ventricle; LV: Left ventricle.

**Figure 4:**
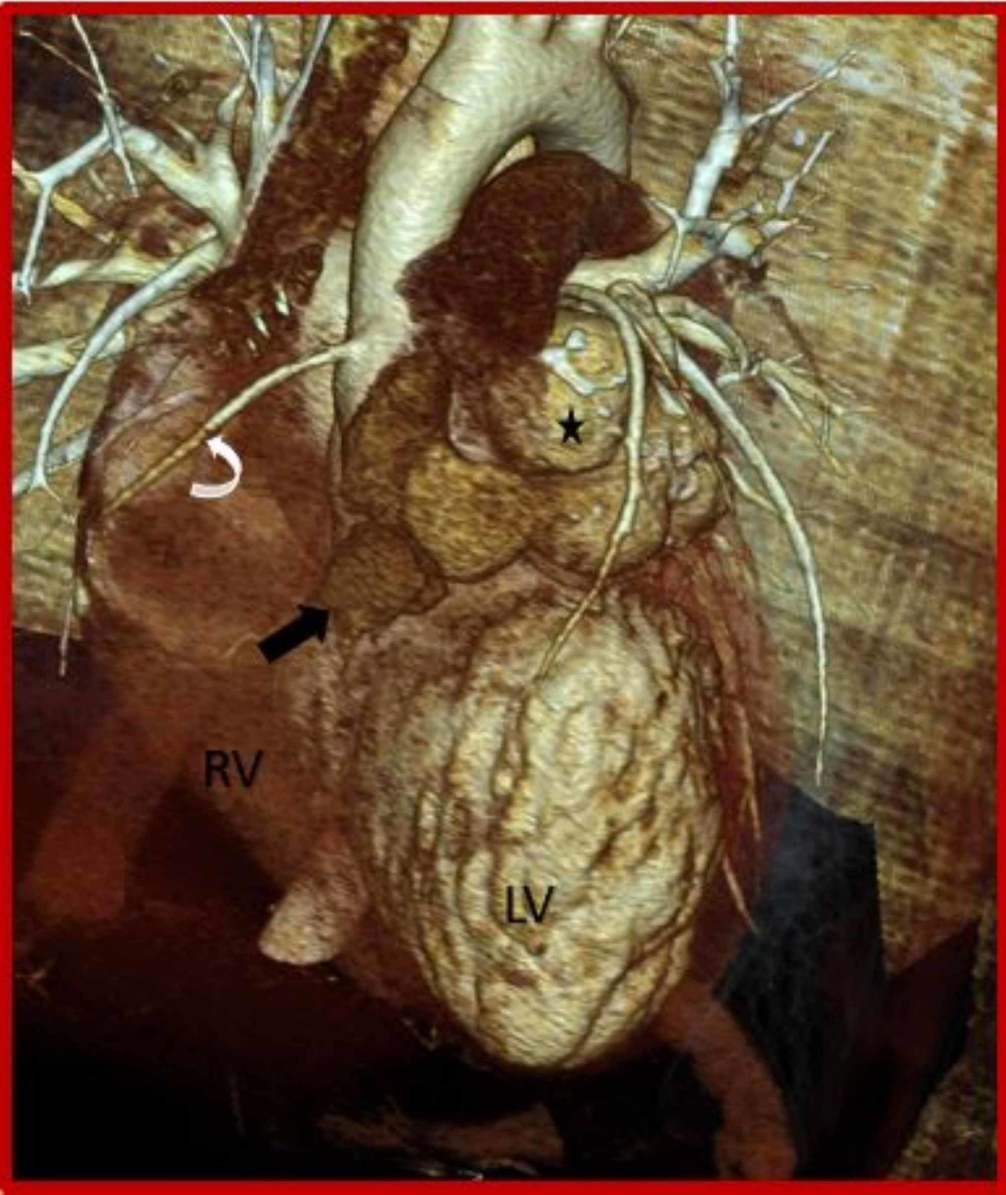
Cardiac CT (3D VRT) demonstrates ruptured left sinus of Valsalva aneurysm (asterisk) dissecting into the interventricular septum (black arrow). CT: Computed tomography; Curved arrow: Right coronary artery; RV: Right ventricle; LV: Left ventricle.

**Figure 5:**
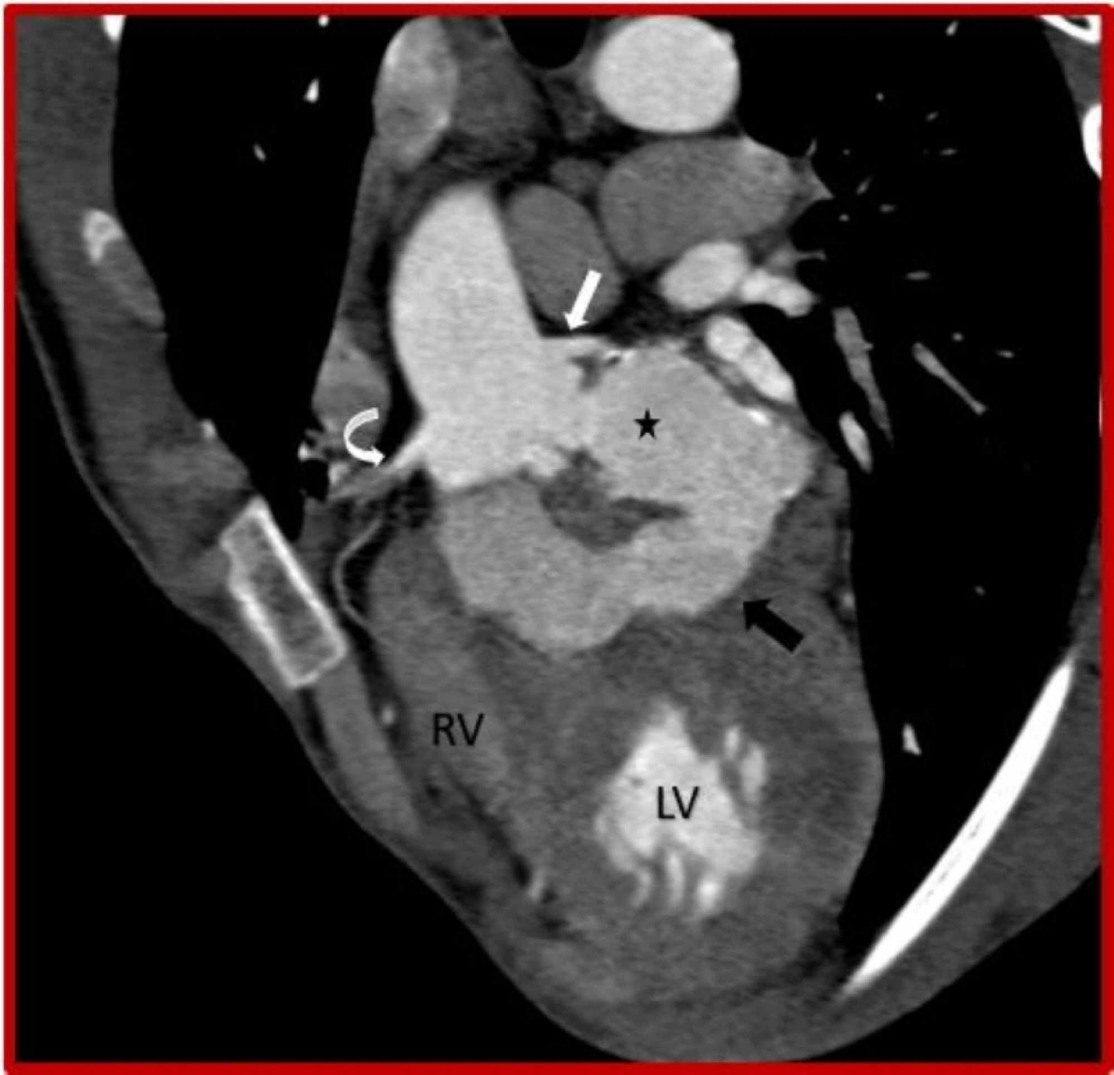
Cardiac CT (coronal oblique view) demonstrates ruptured left sinus of Valsalva aneurysm (asterisk) dissecting into the interventricular septum (black arrow) from the basal to mid septal region. CT: Computed tomography; Curved arrow: Right coronary artery; Straight white arrow: Left coronary artery; RV: Right ventricle; LV: Left ventricle.

The aneurysm was seen in anterior left atrio-ventricular groove dissecting into left antero-lateral myocardium of basal and mid segments of left ventricle. There was a rent in the antero-inferior wall of aneurysm with rupture into the inter-ventricular septum in mid and basal segments (Figures 2-5). No fistulous communication was seen with the ventricular cavity.

The origin of left coronary artery was seen separately from the aneurysmal origin near sino-tubular junction (Figure 5).

**Figure 6:**
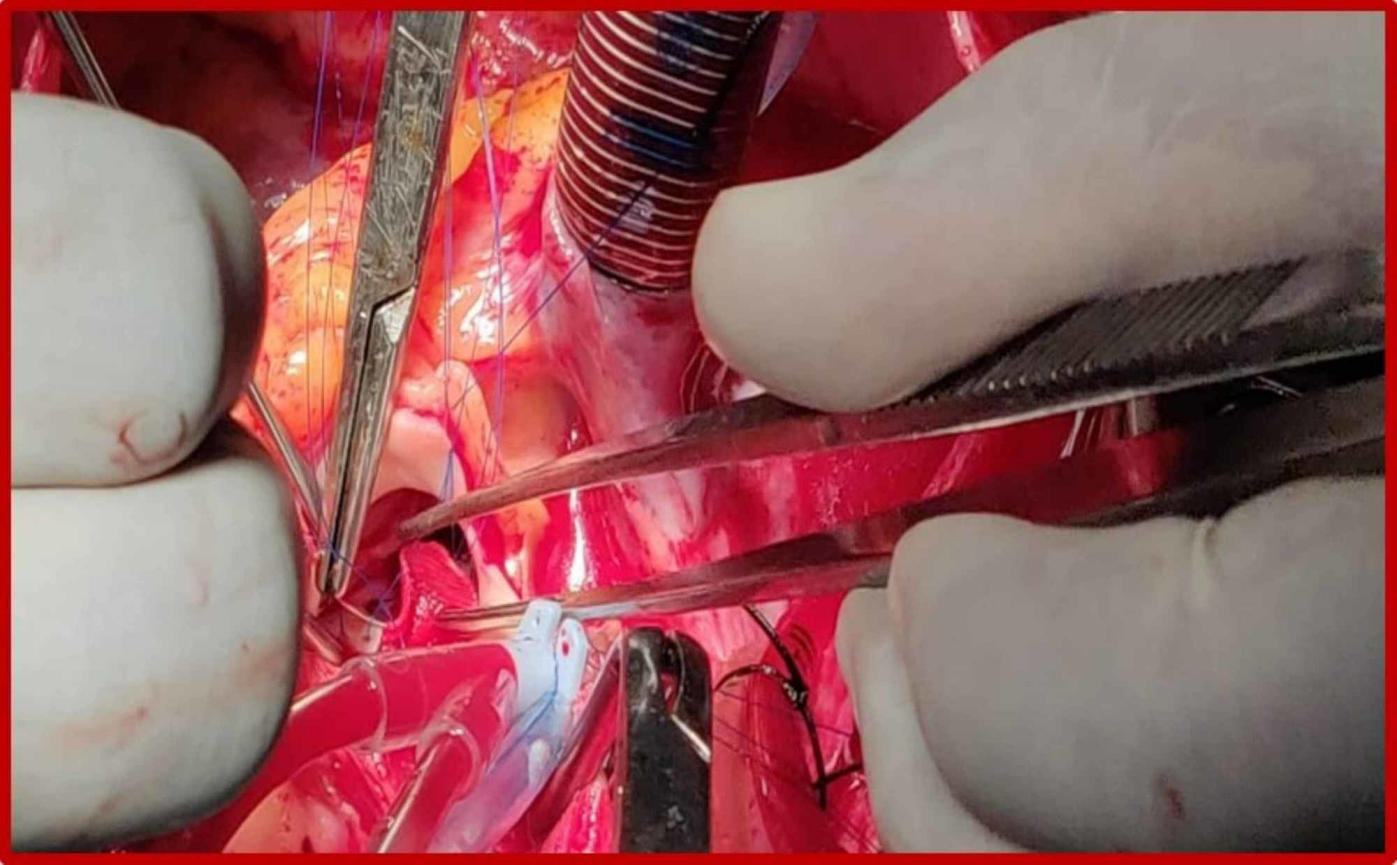
Intraoperative photography demonstrating the aneurysm (arrow) arising from the left sinus of Valsalva.

Additionally, left ventricular concentric hypertrophy was noted. No pericardial thickening or pericardial effusion was seen. The CT findings were later confirmed at surgery and aneurysmal repair was performed (Figure 6).

Case 2

A 35-year-old male patient, who was previously operated with mitral valve replacement five years ago and left ventricular aneurysmal excision with patch repair two years ago, presented with chest discomfort and dryness of throat on exertion. Occasional palpitations were also present.

Echocardiography revealed a left ventricular aneurysm with mild aortic regurgitation and hypokinesia in the basolateral region. The interventricular septum showed a split at the basal segments. The left ventricular ejection fraction was 45%.

**Figure 7:**
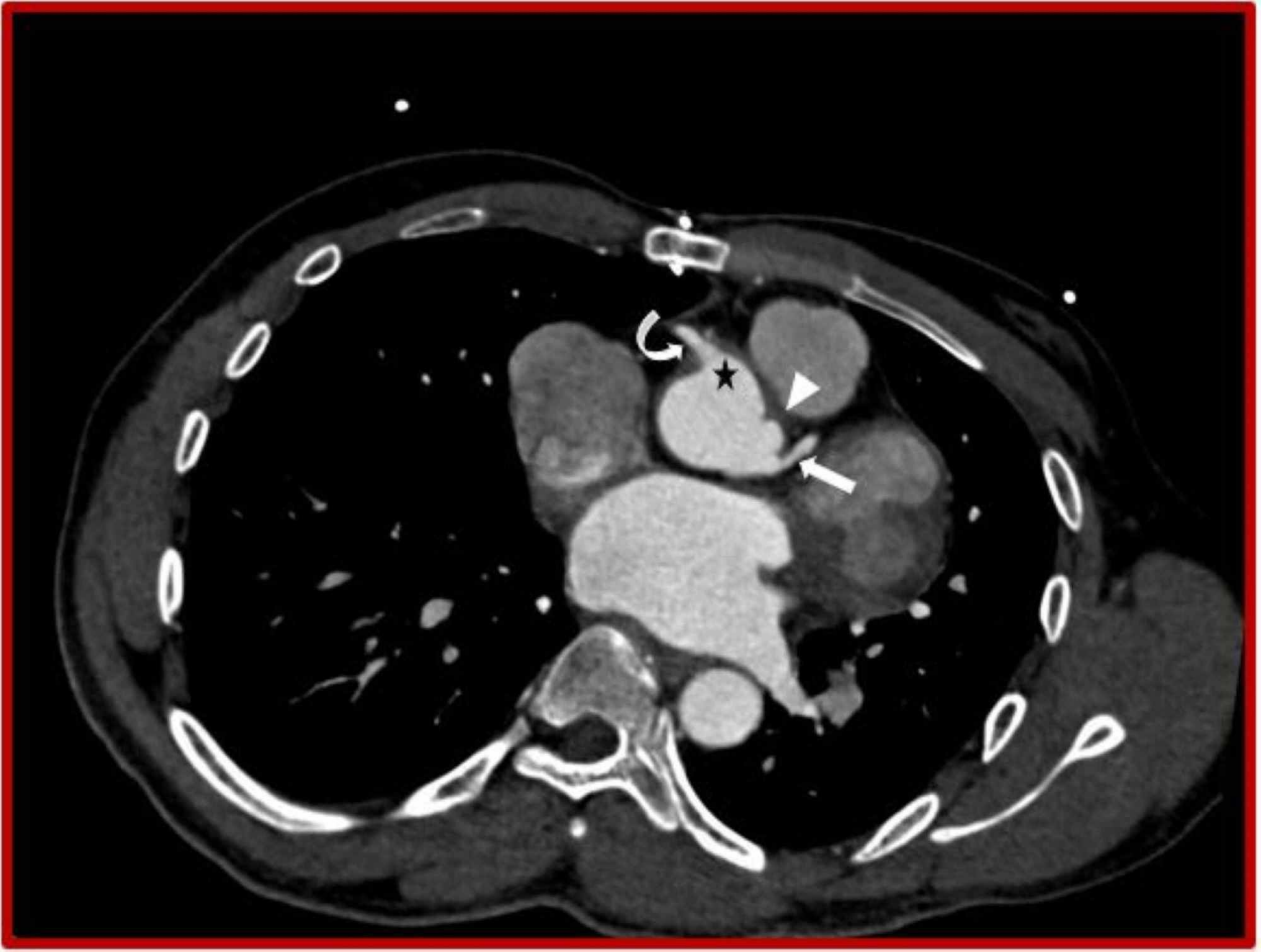
Cardiac CT (Axial view) demonstrates right (asterisk) and left (arrowhead) sinus of Valsalva aneurysms with right (curved arrow) and left (straight arrow) coronary arteries originating from them. CT: Computed tomography.

**Figure 8:**
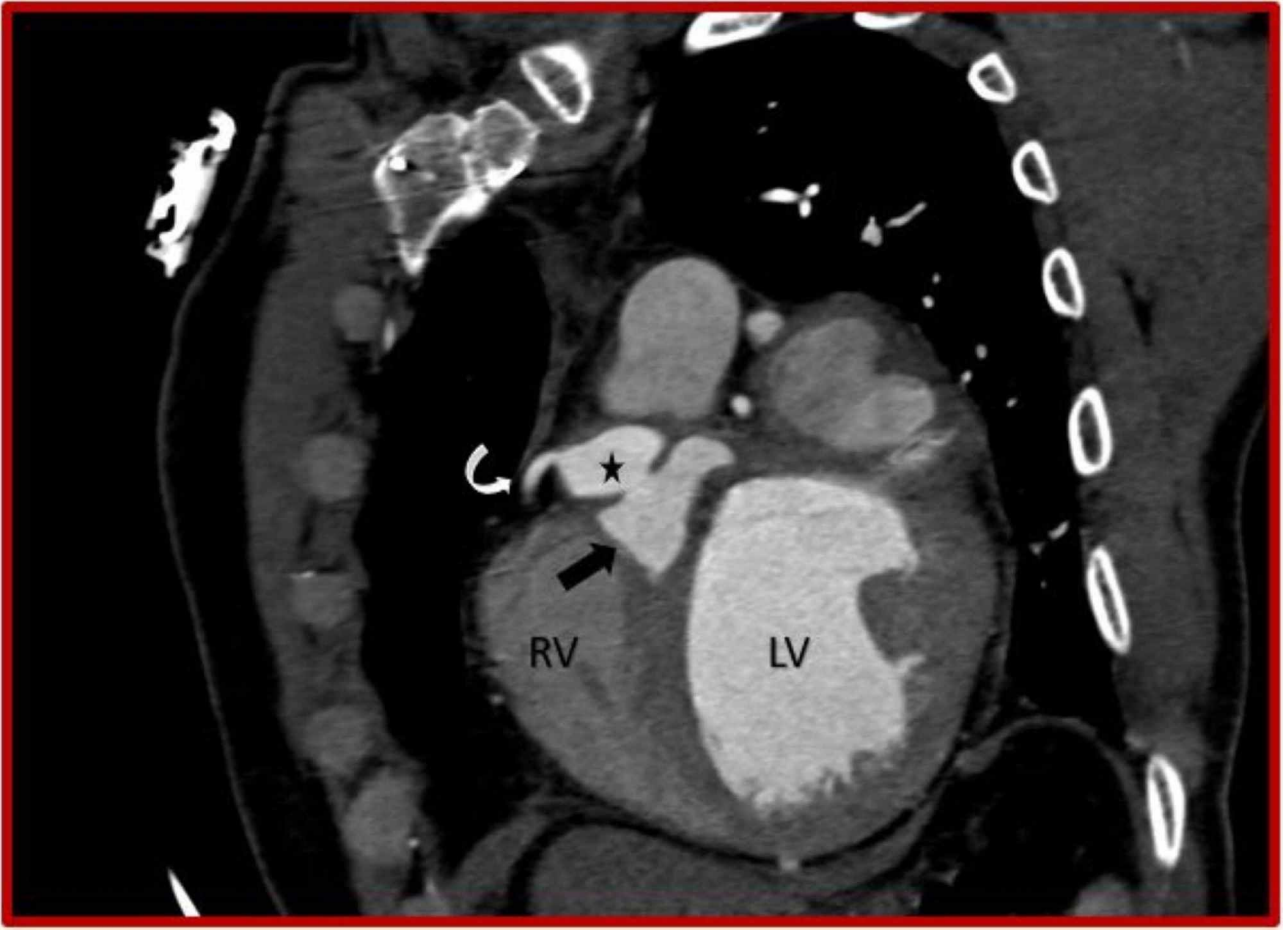
Cardiac CT (coronal oblique view) demonstrates ruptured right sinus of Valsalva aneurysm (asterisk) dissecting into the interventricular septum (black arrow) and right coronary artery (curved arrow) originating from the aneurysm. CT: Computed tomography; RV: Right ventricle; LV: Left ventricle.

**Figure 9:**
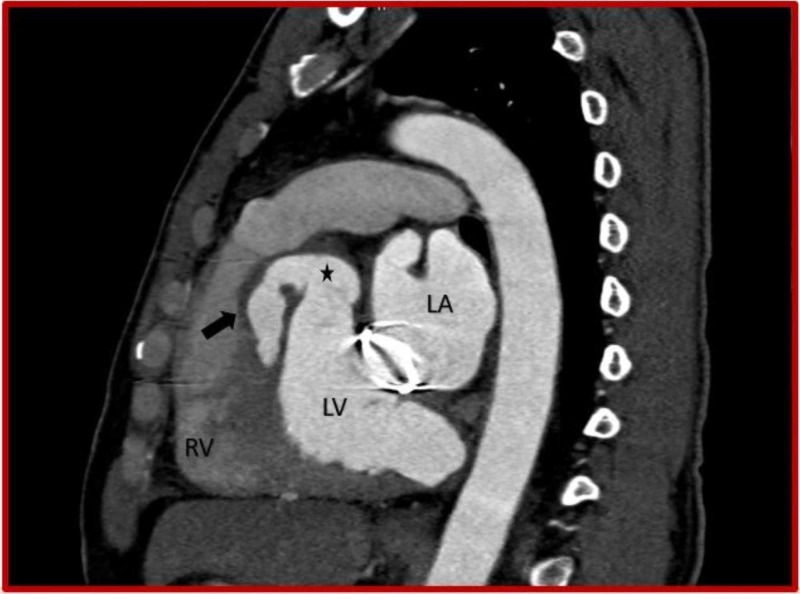
Cardiac CT (sagittal oblique view) demonstrates ruptured right sinus of Valsalva aneurysm (asterisk) dissecting into the interventricular septum (black arrow) and prosthetic mitral valve. CT: Computed tomography; RV: Right ventricle; LA: Left atrium; LV: Left ventricle.

**Figure 10:**
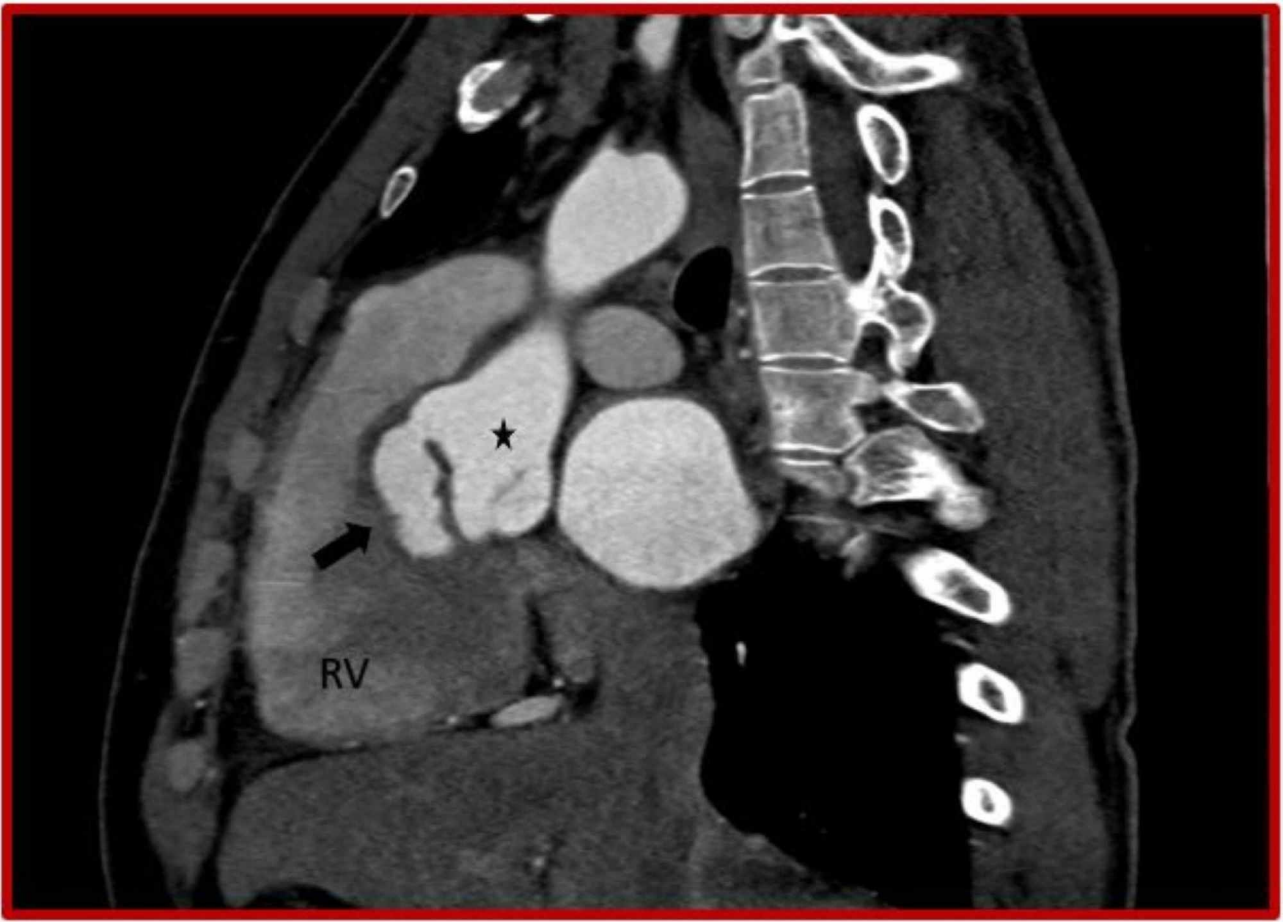
Cardiac CT (sagittal oblique view) demonstrates ruptured right sinus of Valsalva aneurysm (asterisk) dissecting into the interventricular septum (black arrow). CT: Computed tomography; RV: Right ventricle.

**Figure 11:**
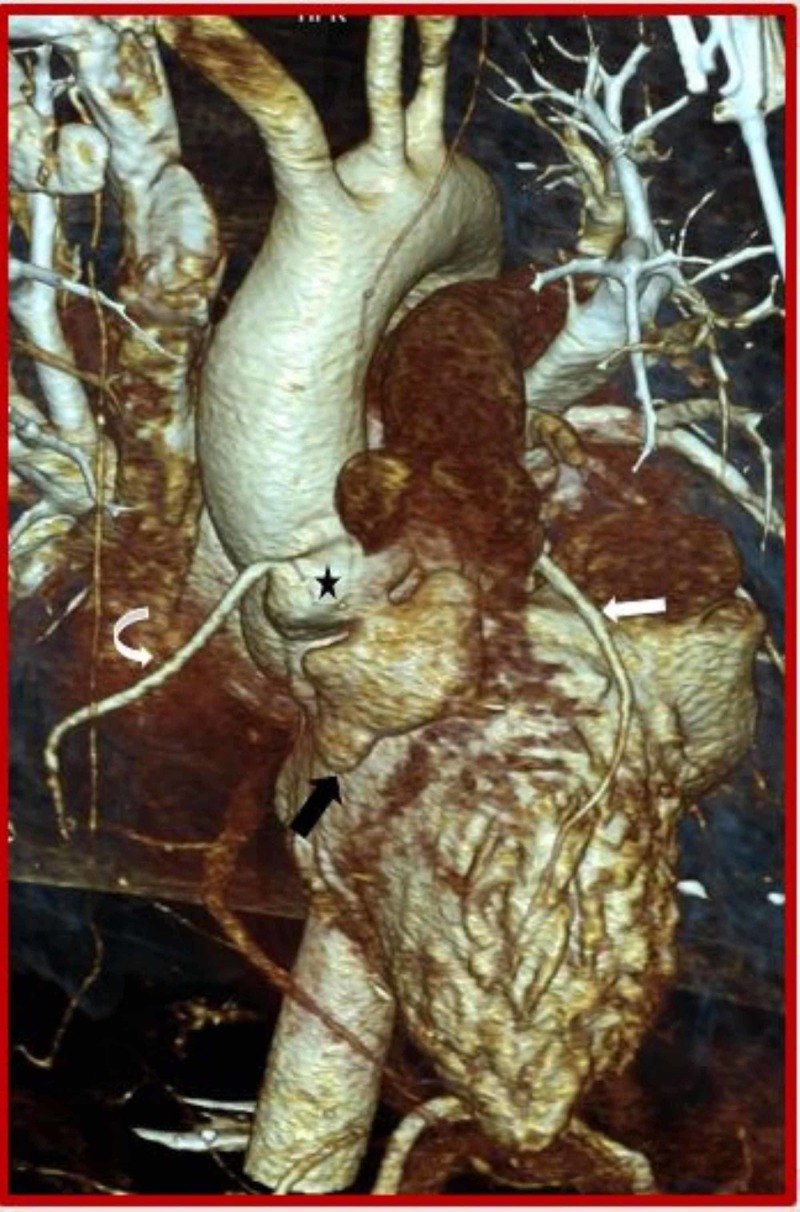
Cardiac CT (3D VRT) demonstrates ruptured right sinus of Valsalva aneurysm (asterisk) dissecting into the interventricular septum (black arrow). CT: Computed tomography; RV: Right ventricle; Curved arrow: Right coronary artery; Straight white arrow: Left coronary artery.

Subsequent evaluation with multi slice cardiac CT revealed a fusiform right coronary sinus of Valsalva aneurysm (Figure 7). This was seen to dissect into the basal and mid part of interventricular septum forming a sinus tract with tiny peripheral ramifications (Figures 8-11). The right coronary artery was arising from the antero-superior part of the aneurysm and was normal in course, caliber and contrast opacification. Right dominance of circulation was noted.

A yet another small left coronary sinus of Valsalva fusiform aneurysm was detected with the origin of left coronary artery from the posterior wall of the aneurysm (Figure 7).

The CT also showed a large wide mouthed bilobed thick walled outpouching arising from the lateral basal wall of the left ventricle suggestive of left ventricular saccular aneurysm. No filling defect was seen in the left ventricle or aneurysm. Left ventricle appeared dilated.

There was mild dilatation of the left atrium. The mitral valve prosthesis was seen in place. No pericardial pathology was observed.

**Figure 12:**
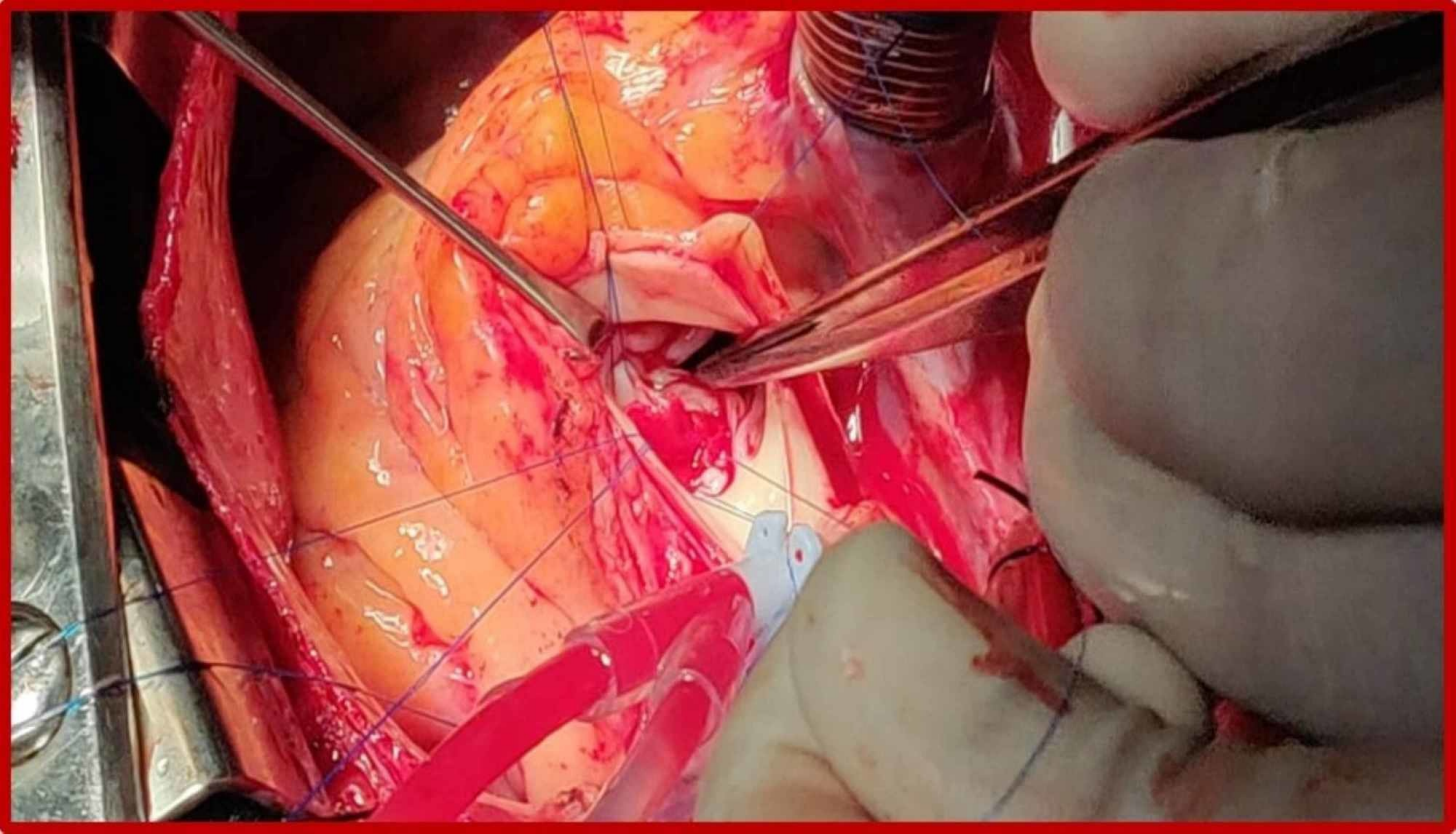
Intraoperative photography demonstrating the aneurysm (arrow) arising from the right sinus of Valsalva.

The patient later underwent an elective surgery for aneurysmal repair and the CT findings were confirmed (Figure 12).

## Discussion

Sinus of Valsalva aneurysm is defined as the dilatation or enlargement of the aortic sinuses located between the aortic valve annulus and the sinotubular junction [2]. The first case in world literature was described by Thurman in 1840 [3]. Their incidence is reported to be 0.15% in open heart surgery patients, with female to male ratio being 3:1 [4]. The incidences of aneurysms arising from the right coronary sinus, noncoronary sinus and left sinus are 70-90%, 10-20% and <5%, respectively [2, 5, 6].

Etiologically, sinus of Valsalva aneurysms can be classified as congenital and acquired. Causes of acquired aneurysms include trauma, tuberculosis, infective endocarditis, Behçet disease, syphilis and atherosclerosis. Congenital aneurysms are associated with bicuspid aortic valve, ventricular septal defects, coronary artery anomalies and aortic regurgitation.

Non-ruptured sinus of Valsalva aneurysm can lead to atrial fibrillation and complete heart block. Rupture of sinus of Valsalva is a rare complication, the consequence of which depends primarily on its location.

Due to rupture of the right and noncoronary Valsalva sinus aneurysms, communication can occur between the aorta and either the right atrium or the right ventricular outflow tract. This in turn can result in a left to right shunt with patients presenting with dyspnea, chest pain, hemodynamic compromise, insidious onset heart failure with fatigue and volume overload. Disrupted aortic root anatomy can cause kinking or compression of coronary ostia with resultant myocardial ischemia [6]. Rupture of left sinus of Valsalva aneurysm is clinically less significant. It causes communication to the left atrium or left ventricular outflow tract [7]. Rupture into pericardial space may lead to cardiac tamponade.

Rupture of the sinus of Valsalva aneurysm into the interventricular septum is an even rarer entity, which is reported to be less than 2% of sinus of Valsalva ruptures [3]. Dissection into the interventricular septum was first reported by Warthen in 1947 [2]. It is also described in few texts as interventricular dissection and is considered an important indication for surgery. Other indications for surgical repair include ventricular outflow tract obstruction and intractable arrhythmias.

ECG-gated multidetector CT (MDCT) can provide images with high spatial resolution. Superior anatomic delineation can be obtained via MDCT due to provision of large field of view and the ability to obtain multiplanar reformations. Another major advantage is simultaneous evaluation of the coronary arteries. CT is, however, inferior to MRI for valvular assessment [8].

## Conclusions

Rupture of sinus of Valsalva aneurysm into the interventricular septum is a rare life-threatening complication. Cardiac CT offers high quality of anatomic detail for precise diagnosis; the multiformat reconstructions and volume rendered techniques provide unparalleled approach for pre-operative planning.
